# Neonatal resuscitation: EN-BIRTH multi-country validation study

**DOI:** 10.1186/s12884-020-03422-9

**Published:** 2021-03-26

**Authors:** Ashish KC, Kimberly Peven, Shafiqul Ameen, Georgina Msemo, Omkar Basnet, Harriet Ruysen, Sojib Bin Zaman, Martha Mkony, Avinash K. Sunny, Qazi Sadeq-ur Rahman, Josephine Shabani, Ram Chandra Bastola, Evelyne Assenga, Naresh P. KC, Shams El Arifeen, Edward Kija, Honey Malla, Stefanie Kong, Nalini Singhal, Susan Niermeyer, Ornella Lincetto, Louise T. Day, Joy E. Lawn, and EN-BIRTH Study Group, Qazi Sadeq-ur Rahman, Ahmed Ehsanur Rahman, Tazeen Tahsina, Sojib Bin Zaman, Shafiqul Ameen, Tanvir Hossain, Abu Bakkar Siddique, Aniqa Tasnim Hossain, Tapas Mazumder, Jasmin Khan, Taqbir Us Samad Talha, Rajib Haider, Md. Hafizur Rahman, Anisuddin Ahmed, Shams Arifeen, Omkar Basnet, Avinash K. Sunny, Nishant Thakur, Regina Gurung, Anjani Kumar Jha, Bijay Jha, Ram Chandra Bastola, Rajendra Paudel, Asmita Paudel, K. C. Ashish, Nahya Salim, Donat Shamba, Josephine Shabani, Kizito Shirima, Menna Narcis Tarimo, Godfrey Mbaruku, Honorati Masanja, Louise T. Day, Harriet Ruysen, Kimberly Peven, Vladimir Sergeevich Gordeev, Georgia R. Gore-Langton, Dorothy Boggs, Stefanie Kong, Angela Baschieri, Simon Cousens, Joy E. Lawn

**Affiliations:** 1 International Maternal and Child Health, Department of Women’s and Children’s Health, Uppsala University, Uppsala, Sweden; 2 Maternal, Adolescent, Reproductive & Child Health (MARCH), London School of Hygiene & Tropical Medicine, Keppel Street, London, WC1E 7HT UK; 3 Florence Nightingale Faculty of Nursing, Midwifery & Palliative Care, King’s College London, London, UK; 4 Maternal and Child Health Division, International Centre for Diarrhoeal Disease Research, Bangladesh (icddr,b), Dhaka, Bangladesh; 5 Department of Health Systems, Impact Evaluation and Policy, Ifakara Health Institute, Dar es Salaam, Tanzania; 6 Ministry of Health and Social Welfare, Dar es Salaam, Tanzania; 7 Research Division, Golden Community, Lalitpur, Nepal; 8 Pokhara Academy of Health Sciences, Pokhara, Nepal; 9 Ministry of Health and Population, Kathmandu, Nepal; 10 Muhimbili University of Health and Allied Sciences (MUHAS), Dar Es Salaam, Tanzania; 11 Society of Public Health Physicians Nepal, Kathmandu, Nepal; 12 Department of Paediatrics, University of Calgary, Calgary, Canada; 13 University of Colorado School of Medicine, Colorado School of Public Health, Aurora, CO USA; 14 Department of Maternal, Newborn, Child and Adolescent Health and Ageing, WHO, Geneva, Switzerland

**Keywords:** Birth, Neonatal resuscitation, Coverage, Quality, Measurement, Validity, Survey, Hospital records, Health management information systems

## Abstract

**Background:**

Annually, 14 million newborns require stimulation to initiate breathing at birth and 6 million require bag-mask-ventilation (BMV). Many countries have invested in facility-based neonatal resuscitation equipment and training. However, there is no consistent tracking for neonatal resuscitation coverage.

**Methods:**

The EN-BIRTH study, in five hospitals in Bangladesh, Nepal, and Tanzania (2017–2018), collected time-stamped data for care around birth, including neonatal resuscitation. Researchers surveyed women and extracted data from routine labour ward registers. To assess accuracy, we compared gold standard observed coverage to survey-reported and register-recorded coverage, using absolute difference, validity ratios, and individual-level validation metrics (sensitivity, specificity, percent agreement). We analysed two resuscitation numerators (stimulation, BMV) and three denominators (live births and fresh stillbirths, non-crying, non-breathing). We also examined timeliness of BMV. Qualitative data were collected from health workers and data collectors regarding barriers and enablers to routine recording of resuscitation.

**Results:**

Among 22,752 observed births, 5330 (23.4%) babies did not cry and 3860 (17.0%) did not breathe in the first minute after birth. 16.2% (*n* = 3688) of babies were stimulated and 4.4% (*n* = 998) received BMV. Survey-report underestimated coverage of stimulation and BMV. Four of five labour ward registers captured resuscitation numerators. Stimulation had variable accuracy (sensitivity 7.5–40.8%, specificity 66.8–99.5%), BMV accuracy was higher (sensitivity 12.4–48.4%, specificity > 93%), with small absolute differences between observed and recorded BMV. Accuracy did not vary by denominator option. < 1% of BMV was initiated within 1 min of birth. Enablers to register recording included training and data use while barriers included register design, documentation burden, and time pressure.

**Conclusions:**

Population-based surveys are unlikely to be useful for measuring resuscitation coverage given low validity of exit-survey report. Routine labour ward registers have potential to accurately capture BMV as the numerator. Measuring the true denominator for clinical need is complex; newborns may require BMV if breathing ineffectively or experiencing apnoea after initial drying/stimulation or subsequently at any time. Further denominator research is required to evaluate non-crying as a potential alternative in the context of respectful care. Measuring quality gaps, notably timely provision of resuscitation, is crucial for programme improvement and impact, but unlikely to be feasible in routine systems, requiring audits and special studies.

**Supplementary Information:**

The online version contains supplementary material available at 10.1186/s12884-020-03422-9.

## Key findings

**Table:** 

**What is known and what is new about this study?**	
• Neonatal resuscitation programmes are being scaled up globally, yet coverage of resuscitative interventions is not routinely tracked. Resuscitation coverage and quality measures have not yet been validated in either population-based surveys or routine facility registers.	
• Challenges exist for measurement of resuscitation coverage indicators:	
*° Numerator:* Which action during clinical resuscitation (e.g. stimulation or bag-mask-ventilation [BMV]) is both measurable and valid?	
*° Denominator:* What is measurable and useful (e.g. live births plus fresh stillbirths or non-breathing, or non-crying babies)?	
• EN-BIRTH is the first observational study (> 23,000 births) to assess validity of neonatal resuscitation coverage measurement, in both exit survey of women’s report and routine register records. Using time-stamped data, we analysed coverage and quality of neonatal resuscitation in five hospitals in Bangladesh, Nepal, and Tanzania.	
**Survey — what did we find and what does it mean?**	
• *Numerator options*: Survey-reported coverage of BMV (0.3–1.9%) markedly under-estimated observed coverage (0.7–7.1%). BMV had low sensitivity (< 21%) and high specificity (> 98%). Newborn stimulation was reported by < 3% of women, very much lower than observed coverage (5.2–21.0%).	
• *Denominator options*: Crying at birth had low “don’t know” responses (< 3%) in exit survey. Compared to observed crying within a minute of birth, sensitivity was high (> 95%); however, specificity was low (< 22%). Survey-reported BMV coverage validity was consistently low for all denominators assessed.	
**Register — what did we find and what does it mean?**	
• *Numerator options:* Stimulation and BMV were recorded by 4 of 5 labour ward registers, yet accuracy varied between hospitals even with the same register design. BMV sensitivity ranged from 12.4–48.4% and specificity was high (> 93%). For stimulation, sensitivity was low at 7.5–40.8% and specificity was more variable (range 66.8–99.5).	
• *Denominators:* Livebirths and fresh stillbirths were recorded in all registers. The “non-crying/non-breathing” combined denominator was only in the Bangladesh registers and could not be validated. Register-recorded BMV coverage was consistent whichever denominators was applied.	
**Gap analysis for quality of care and measurement**	
• Most newborns (71.4–94.7%) who did not respond to stimulation did receive BMV, but only 1% within the recommended 1 min after birth.	
**What next and research gaps?**	
• Population-based surveys are not likely to be useful for measuring neonatal resuscitation coverage, given low validity of exit-survey report. Additionally, household surveys would be underpowered since resuscitation is required by a small proportion of babies.	
• Routine hospital registers have potential to track resuscitation coverage indicators, but implementation research is needed to standardise design and processes, including data flow to Health Management Information Systems. BMV is the most accurate numerator, true denominator measurement is complex and requires more research, including assessment of non-crying.	
• Data use with feedback loops and support to frontline healthcare workers could help improve data quality and quality of care. Local clinical quality improvement and special studies are important to reduce quality gaps, particularly for timely BMV, and help meet global goals to end preventable deaths.	

## Background

Annually, 7–14 million newborns (5–10%) are estimated to require stimulation to initiate breathing at birth and 6 million newborns require bag-mask-ventilation (BMV) [1, 2]. Intrapartum-related events (previously termed “birth asphyxia”) are a leading cause of neonatal mortality, accounting for 11% of under-five deaths [2, 3]. Such intrapartum-related events can cause stillbirths just before birth and neonatal deaths just after. The majority (> 84%) of stillbirths are in low- and middle-income countries (LMICs) and an estimated 50% are intrapartum [4, 5]. Resuscitation is recommended for all babies who do not breathe after birth since live births may be misclassified as stillbirths [6, 7]. Meeting Sustainable Development Goal (SDG) targets by 2030 for ending preventable neonatal deaths requires universal coverage of high quality care around birth for women and their babies, including resuscitation for those who do not breathe at birth [8, 9]. Globally ~ 80% of births are now in facilities [10], with many LMICs scaling up neonatal resuscitation programs [11–13]. However, lack of measurement for coverage and quality of neonatal resuscitation impedes tracking of progress [14].

The definition of coverage requires a *numerator* capturing the intervention (or a component) divided by a target *denominator* regarding clinical need. A good indicator may not include all of the clinical intervention but should “indicate” well and also not incentivise undesirable practices. Resuscitation coverage measurement has specific challenges. Clinical algorithms have multiple actions that could be used as numerators, notably: stimulation of the baby or the action of BMV. Suction is indicated for some babies, but inappropriate suctioning can be harmful, thus should be avoided for a measurement focus [15].

**Fig. 1:**
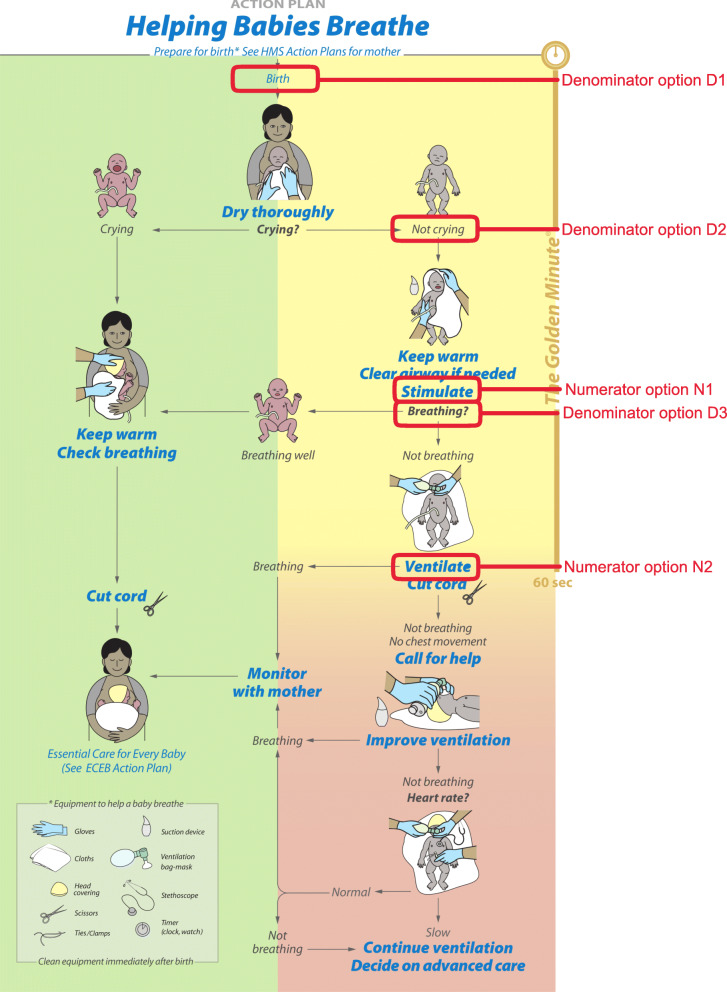
Helping Babies Breathe algorithm decision points to measure neonatal resuscitation coverage

Resuscitation algorithms start at birth for all babies, including fresh stillbirths, being dried and assessed for crying or breathing. WHO guidance on basic resuscitation focuses on the baby who is not breathing spontaneously or is depressed [16]. A global partnership called “Helping Babies Breathe,” (HBB) widely used for neonatal resuscitation training in LMICs, uses crying during thorough drying as a rapid and objective assessment, then evaluating breathing (Fig. 1) [17]. In line with WHO guidelines, if the baby is not crying and not breathing, then stimulation is provided to improve or initiate breathing, and clearing of the airway if it is blocked with secretions. If the baby is not breathing after these actions BMV should begin within 1 min of birth.

Most data on maternal and newborn health care coverage in LMICs relies on population-based surveys, notably Demographic and Health Surveys (DHS) and Multiple Indicator Cluster Surveys (MICS), none of which capture neonatal resuscitation. Routine facility data are currently an underutilised source for neonatal resuscitation coverage for routine Health Management Information Systems (HMIS). Interventions around the time of birth are typically recorded in one or more facility documents: individual patient records, labour and delivery ward registers, and intervention-specific registers (e.g., neonatal resuscitation register) [18]. Previous research has demonstrated availability of some neonatal resuscitation data in routine labour ward registers [19, 20]. Use of HMIS data aggregated from registers is impeded by concerns regarding data quality [21], but to date no validation studies have been undertaken regarding either survey or routine register data for neonatal resuscitation coverage indicators.

The *Every Newborn* Action Plan, agreed by all 195 United Nations member states, includes an ambitious measurement improvement roadmap [9] to validate coverage indicator measurement for care and outcomes around the time of birth. The *Every Newborn*–Birth Indicators Research Tracking in Hospitals (EN-BIRTH) study was undertaken in three countries (Tanzania, Bangladesh, and Nepal) and aimed to assess validity of measurement of selected newborn and maternal indicators for routine facility-based tracking of coverage, quality of care, and outcomes [22].

## Objectives

This paper is part of a supplement based on the EN-BIRTH multi-country validation study, *‘Informing measurement of coverage and quality of maternal and newborn care’*, and focuses on neonatal resuscitation measurement with four objectives:

1. **Assess NUMERATOR accuracy/validity** for neonatal resuscitation coverage indicator (stimulation and BMV) measurement by exit survey of women’s report and routine labour ward registers compared to direct observation (gold standard).
2. **Compare DENOMINATOR options** for resuscitation coverage measurement: including all births (except macerated stillbirths), non-crying babies and non-breathing babies.
3. **Analyse GAPS in coverage, quality of care and measurement** in relation to recommendations, notably timely initiation of BMV.
4. **Evaluate BARRIERS AND ENABLERS** to routine labour ward register recording for resuscitation regarding register design, filling, and use.

## Methods

**Fig. 2:**
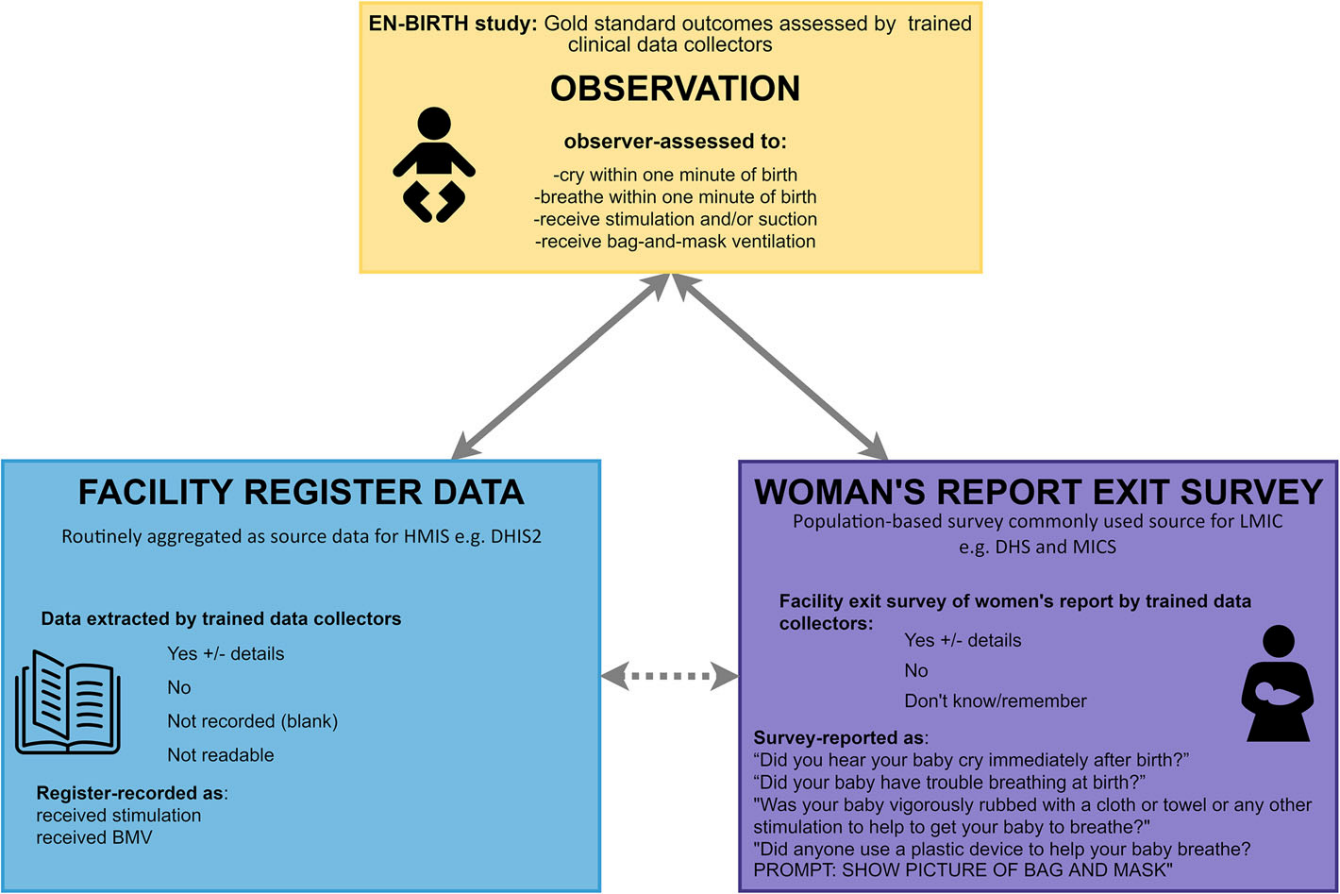
Neonatal resuscitation validation design, EN-BIRTH study. EN-BIRTH validation design comparing observation gold standard with register-recorded and women’s report on exit survey; EN-BIRTH data collection tools (observation checklist, register data extraction tool and exit survey tool) are published separately [22]

EN-BIRTH was an observational, mixed methods study comparing data from clinical observers (gold standard) to survey-reported and register-recorded coverage of perinatal care and outcomes (Fig. 2). Detailed information regarding the research protocol, methods, and analysis has been published separately [22, 23]. Data were collected from July 2017–July 2018 in five public CEmONC hospitals in three high mortality burden countries: Maternal and Child Health Training Institute, Azimpur and Kushtia General Hospital in Bangladesh (BD); Pokhara Academy of Health Sciences in Nepal (NP); Temeke Regional Hospital and Muhimbili National Referral Hospital in Tanzania (TZ). (Additional file 1). Baseline health facility assessments established that all five hospitals had capacity to resuscitate newborns. Resuscitation guidelines used in all five hospitals were based on HBB [17]. Participants were consenting women admitted in labour for care around birth. Exclusion criteria included imminent birth and no fetal heart beat heard on admission. Clinically trained researchers observed participants 24 h per day and recorded data on the baby’s condition at birth (e.g., crying/breathing) and care (e.g., stimulation and BMV). The observers received refresher training in HBB as part of their clinical observation training before the study started [22]. Data were collected with a custom-built android tablet-based application, including timestamps for observations. Research data collectors interviewed women after discharge before exit from hospital regarding their baby’s condition after birth and care received. Resuscitation and outcome data were extracted from routine hospital registers. Metadata definitions of selected indicator options for validity testing are shown in Additional file 2. To determine the reliability of the observational data (gold standard) supervisors duplicated observation (and register data extraction) for a subset of 5% to calculate Cohen’s Kappa coefficients. Health workers and data collectors were interviewed about barriers and enablers to use of routine registers in recording of perinatal care and outcomes.

Results are reported in accordance with STROBE Statement checklists for cross-sectional studies (Additional file 3). Quantitative analysis was undertaken using R version 3.6.1 [24].

### Objective 1: Numerator for indicator measurement validation

Livebirths and fresh stillbirths (hereafter referred to as “newborns”), were considered to require initial assessment for resuscitation, whilst macerated stillbirths were excluded. We explored accuracy of two possible numerator options N1) Stimulation and N2) BMV in both survey and register data compared to observation data.

In exit surveys, where a woman reported her newborn had difficulty breathing at birth, she was asked about resuscitation practices. In line with common survey indicator reporting, where women replied, “don’t know” we considered the survey-reported stimulation/BMV response as “no”.

We compared observed coverage (gold standard) of stimulation and BMV to survey-reported and register-recorded coverage. We calculated absolute differences between measured coverage (survey or register) and observed coverage to understand under- or over-estimation at the population level. Using two-way tables, we calculated individual-level validity statistics: sensitivity, specificity, and percent agreement ((true positive + true negative)/total) of register-recorded and survey-reported BMV coverage to measure observed coverage. Area under the curve, inflation factor, positive predictive value, and negative predictive value were also calculated. All calculations were stratified by hospital with 95% confidence intervals. Pooled results for validity analyses were calculated using random effects meta-analysis, presented with i^**2**^, τ^**2**^, and heterogeneity statistic (Q).

### Objective 2: Denominator comparisons

We explored neonatal resuscitation coverage measurement using three possible denominator options: D1) all newborns (total births excluding macerated stillbirths), D2) newborns not crying within the first minute after birth and D3) newborns not breathing within the first minute after birth.

We compared these denominators using validity ratios (measured:observed coverage), similar to verification ratios in data quality review methods [25], for survey-reported and register-recorded BMV coverage. Validity ratios > 1 show overestimation of survey-reported or register-recorded coverage compared to observed, while ratios < 1 show underestimation. Results were heat-mapped using standard data quality review cut-offs (over/underestimate by 0–5%, 6–10%, 11–15%, 16–20 and > 20%).

### Objective 3: Gap analysis for coverage and quality of care, and measurement

We examined gaps in coverage and timely neonatal resuscitation amongst a subset of newborns with a clinical need for resuscitation within 1 min of birth. These newborns were not breathing in the first minute after birth and did not respond to stimulation (or suction when performed). For this (A) eligible population subset, we analysed four gaps for neonatal resuscitation: (B) coverage gap for BMV, (C) quality of care gap between *any* BMV coverage, and timely coverage (within 1 min), (D) measurement gap for survey-report, and (E) measurement gap for register-record.

### Objective 4: Barriers and enablers to routine recording

Qualitative data collection tools for focus group discussions and in-depth interviews were informed by the Performance of Routine Information System Management Series (PRISM) conceptual framework [26]. Detailed qualitative methods and overall results are available in an associated paper [27]. A purposive sample of nurses, midwives, doctors, and EN-BIRTH data collectors from each of the five hospitals participated. Analysis identified themes based on three domains: register design, filling, and use [26]. In addition, respondents were asked questions regarding the order in which resuscitation is documented in registers, patient notes, and other documents as well as how long after resuscitation is documentation entered in the labour ward register. This paper presents emerging themes regarding recording of neonatal resuscitation.

## Results

**Fig. 3:**
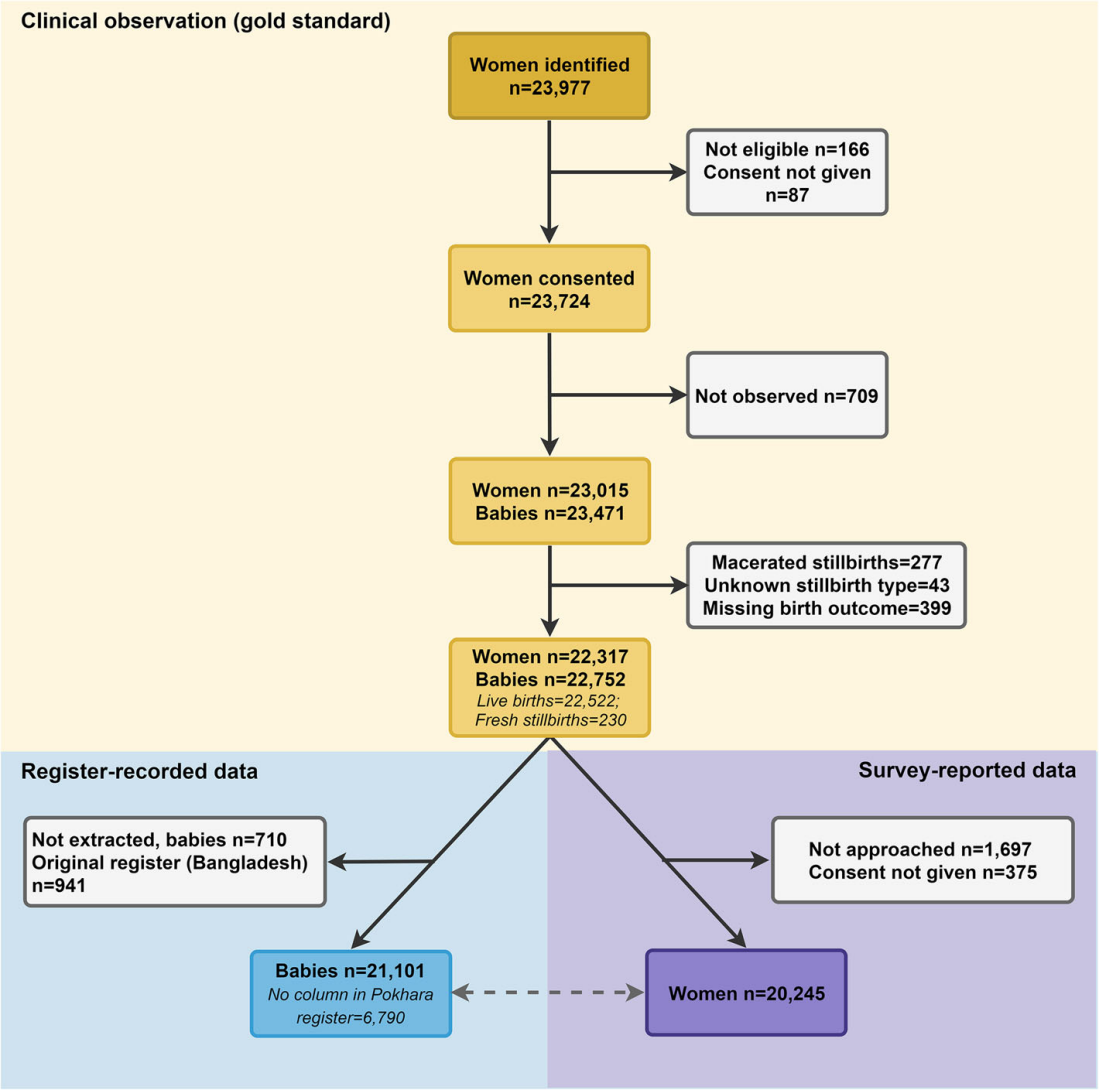
Flow diagram of cases for neonatal resuscitation analysis, EN-BIRTH study (*n* = 22,752)

Among 23,811 eligible women across the five participating hospitals, 23,724 consented to participate (Fig. 3). Among 23,471 observed births, 22,752 were live births (22,522) or fresh stillbirths (230). Data extraction was completed for 21,101 newborns (92.7%), and exit surveys were conducted with 20,245 women (90.7%). Reasons for women’s non-participation in exit survey included refusals and exit from facility prior to research team approach. Table 1 shows characteristics of newborns in the EN-BIRTH study sample, by hospital. Overall, 98.7% were alive at discharge from labour and delivery, 1% were fresh stillbirths, and less than 1% were born alive but died on the labour ward. Nearly one-third of births (29.5%) were by caesarean section, highest (73.6%) in Azimpur BD.

**Table 1:** Characteristics of babies and women, EN-BIRTH study (*n* = 22,752 births)

	Bangladesh		Nepal	Tanzania		All sites^**a**^
	Azimpur Tertiary	Kushtia District	Pokhara Regional	Temeke Regional	Muhimbili National	
	n (%)	n (%)	n (%)	n (%)	n (%)	n (%)
**a) Total babies observed**	2903	2352	7211	6702	3584	22,752
**Birth outcome - Live Birth**	2896 (99.7)	2308 (98.2)	7175 (99.5)	6634 (99)	3509 (97.9)	22,522 (99.0)
**Newborn condition at L&D discharge**						
Alive	2895 (99.7)	2302 (97.9)	7171 (99.4)	6606 (98.6)	3490 (97.4)	22,464 (98.7)
Fresh stillbirth	7 (0.2)	44 (1.9)	36 (0.5)	68 (1.0)	75 (2.1)	230 (1.0)
Neonatal death	1 (0.0)	6 (0.3)	4 (0.1)	28 (0.4)	19 (0.5)	58 (0.3)
**Mode of birth**						
Normal vaginal birth	766 (26.4)	1369 (58.2)	5812 (80.6)	6213 (92.7)	1513 (42.2)	15,673 (68.9)
Vaginal breech/ Vacuum/ Forceps	1 (0.0)	0 (0.0)	342 (4.7)	10 (0.1)	9 (0.3)	362 (1.6)
Caesarean Section	2136 (73.6)	983 (41.8)	1057 (14.7)	478 (7.1)	2060 (57.5)	6714 (29.5)
**Birthweight of baby < 2500 g**	353 (12.1)	471 (20.0)	840 (11.7)	480 (7.2)	938 (26.2)	3082 (13.5)
**Sex Female/Girl baby**	1439 (49.6)	1143 (48.6)	3329 (46.2)	3229 (48.4)	1760 (49.5)	10,900 (48.1)
**b) Total women observed**^**b**^	2879	2309	7145	6584	3400	22,317
**Women’s age**						
< 18 years	25 (0.9)	2 (0.1)	305 (4.3)	25 (0.4)	7 (0.2)	364 (1.6)
18–19 years	467 (16.2)	189 (8.2)	800 (11.2)	752 (11.4)	152 (4.5)	2360 (10.6)
20–24 years	1150 (39.9)	901 (39)	2989 (41.8)	2263 (34.4)	687 (20.2)	7990 (35.8)
25–29 years	856 (29.7)	714 (30.9)	2051 (28.7)	1655 (25.1)	1087 (32.0)	6363 (28.5)
30–34 years	294 (10.2)	358 (15.5)	790 (11.1)	1117 (17.0)	883 (26.0)	3442 (15.4)
35+ years	87 (3.0)	145 (6.3)	210 (2.9)	772 (11.7)	584 (17.2)	1798 (8.1)
**Women’s education**						
No education	37 (1.3)	75 (3.2)	259 (3.6)	196 (3.0)	63 (1.9)	630 (2.8)
Primary incomplete	110 (3.8)	117 (5.1)	244 (3.4)	76 (1.2)	40 (1.2)	587 (2.6)
Primary complete	333 (11.6)	329 (14.2)	289 (4.0)	28 (0.4)	4 (0.1)	983 (4.4)
Secondary incomplete	976 (33.9)	917 (39.7)	1589 (22.2)	3956 (60.1)	1224 (36.0)	8662 (38.8)
Secondary complete or higher	1263 (43.9)	837 (36.2)	4381 (61.3)	2295 (34.9)	2055 (60.4)	10,831 (48.5)
Missing	160 (5.6)	34 (1.5)	383 (5.4)	33 (0.5)	14 (0.4)	624 (2.8)
**Parity**						
Nullipara	1333 (46.3)	981 (42.5)	4272 (59.8)	2848 (43.3)	1290 (37.9)	10,724 (48.1)
Multipara	1493 (51.9)	1323 (57.3)	2866 (40.1)	3723 (56.5)	2106 (61.9)	11,511 (51.6)
Missing	53 (1.8)	5 (0.2)	7 (0.1)	13 (0.2)	4 (0.1)	82 (0.4)

^a^Individually weighted

^b^Data were collected from women’s registration and survey report

**Fig. 4:**
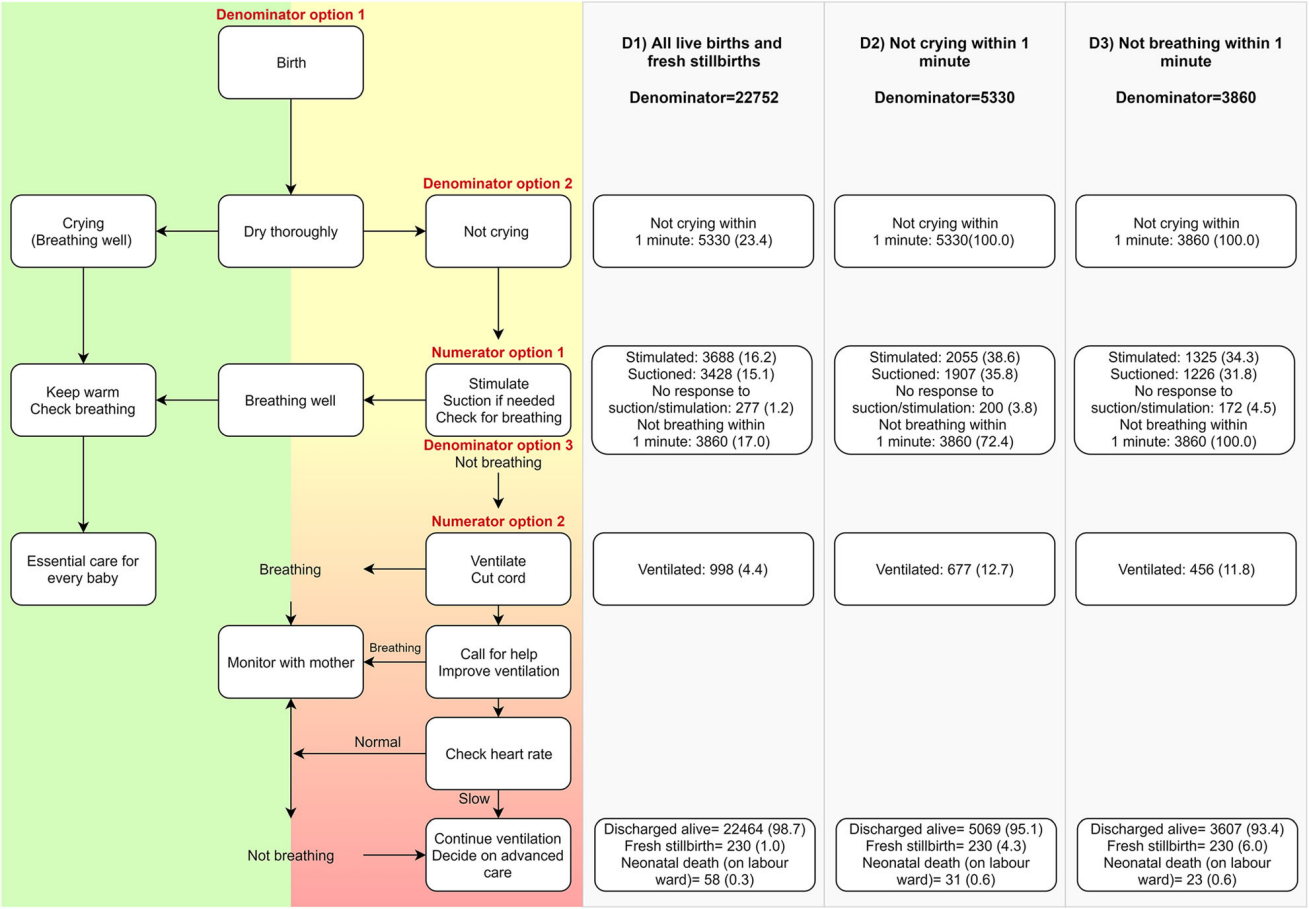
Neonatal resuscitation numerators and denominators, EN-BIRTH study (individually weighted, observation data, *n* = 22,752)

Among 22,752 newborns (denominator option D1), 3688 (16.2%) were stimulated (numerator option N1) and 998 (4.4%) received BMV (numerator option N2) (Fig. 4). Within the first minute after birth, 5330 were observed as non-crying (denominator option D2), and among these 3860 were also observed as non-breathing (denominator option D3).

### Assessing biases in the data

Duplicate case observation inter-rater reliability showed substantial agreement (> 0.71) for resuscitation elements (Additional file 4). Register extraction agreement was lower and varied greatly between sites, ranging from − 0.035 to 0.939.

### Objective 1: Numerator for indicator measurement validation

#### Numerator option 1: stimulation

Observed coverage of stimulation ranged from 5.2% in Azimpur BD to 21.0% in Muhimbili TZ. Survey-report gave large underestimates for stimulation with survey-reported coverage ranging from 0.6–2.2%. Sensitivity was very low (< 14%) while specificity was high (> 98%) (Table 2; additional validity details in Additional file 5 and Additional file 6).

**Table 2:** Individual-level validation in exit surveys and registers for stimulation at birth indicator, EN-BIRTH study (n = 22,752)

	Bangladesh	Bangladesh	Nepal	Tanzania	Tanzania	All sites
	Azimpur Tertiary	Kushtia District	Pokhara Regional	Temeke Regional	Muhimbili National	Pooled (random effects)
**Stimulation - Survey reported - live births + fresh stillbirths**						
Observer coverage %	5.2 (4.4,6.1)	10.2 (9.0,11.5)	20.6 (19.6,21.5)	15.8 (15.0,16.7)	21 (19.7,22.4)	13.9 (8.7,20.1)
Survey reported coverage %	0.7 (0.4,1.1)	2.2 (1.7,2.9)	0.7 (0.5,0.9)	0.6 (0.5,0.9)	1.4 (1.0,1.9)	1.0 (0.6,1.6)
“Don’t know” responses %	3.3 (2.7,4.1)	0.8 (0.5,1.3)	1.4 (1.1,1.7)	0.3 (0.1,0.4)	4 (3.3,4.9)	1.7 (0.6,3.3)
Sensitivity % (95% CI)	7.2 (3.5,12.9)	13.2 (9.1,18.5)	2.5 (1.7,3.6)	3.6 (2.4,5.2)	3.1 (1.7,5.1)	4.8 (2.5,7.8)
Specificity % (95% CI)	99.7 (99.3,99.8)	99 (98.4,99.4)	99.8 (99.7,99.9)	99.8 (99.6,99.9)	99 (98.5,99.4)	99.5 (99.2,99.8)
Percent agreement (TN + TP/n) %	95.0	90.6	80.2	86.8	81.6	86.9 (80.9,92.0)
**Stimulation - Register recorded - live births and fresh stillbirths**						
Observer coverage %	5.2 (4.4,6.1)	10.2 (9.0,11.5)	–	15.8 (15.0,16.7)	21.0 (19.7,22.4)	12.4 (6.7,19.6)
Register recorded coverage %	0.8 (0.5,1.3)	7.7 (6.6,9.0)	–	17.4 (16.5,18.3)	34.8 (33.3,36.4)	12.3 (2.3,28.7)
Not recorded %	98.7 (98.1,99.1)	91.8 (90.5,93.0)	–	20.0 (19.0,21.0)	30.8 (29.2,32.3)	65.9 (20.7,97.8)
Not readable %	0.5 (0.3,0.9)	0.4 (0.2,0.9)	–	0.3 (0.2,0.5)	0.3 (0.1,0.5)	0.4 (0.3,0.5)
Sensitivity % (95% CI)	7.5 (3.3,14.2)	15.2 (10.5,21.0)	–	39.5 (36.5,42.6)	40.8 (37.2,44.5)	24.8 (13.3,38.5)
Specificity % (95% CI)	99.5 (99.1,99.8)	93.1 (91.8,94.2)	–	86.8 (85.9,87.7)	66.8 (65,68.5)	89.4 (73.8,98.5)
Percent agreement (TN + TP/n) %	95.1	85.6	–	79.3	61.3	81.9 (67.4,92.9)

Full denominator details presented in Additional file 14

**Fig. 5:**
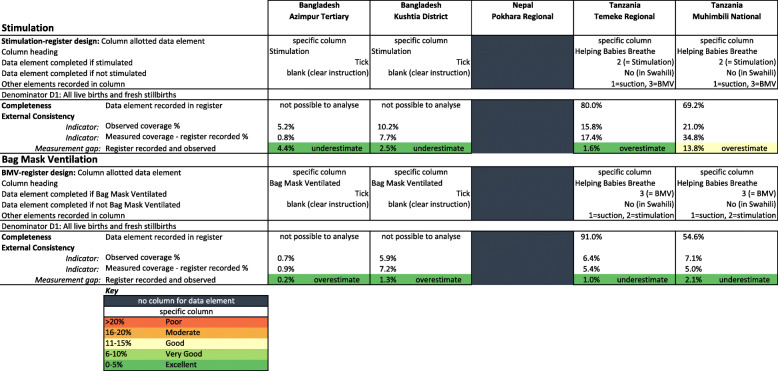
Hospital register design and completeness for stimulation and bag-mask-ventilation, EN-BIRTH study (*n* = 22,752)

Register-recorded coverage (0.8–34.8%) underestimated coverage in the Bangladesh hospitals and overestimated coverage in the Tanzania hospitals (Fig. 5). While sensitivity was low (< 41%), specificity was high across most sites (66.8–99.5%).

#### Numerator option 2: BMV

**Fig. 6:**
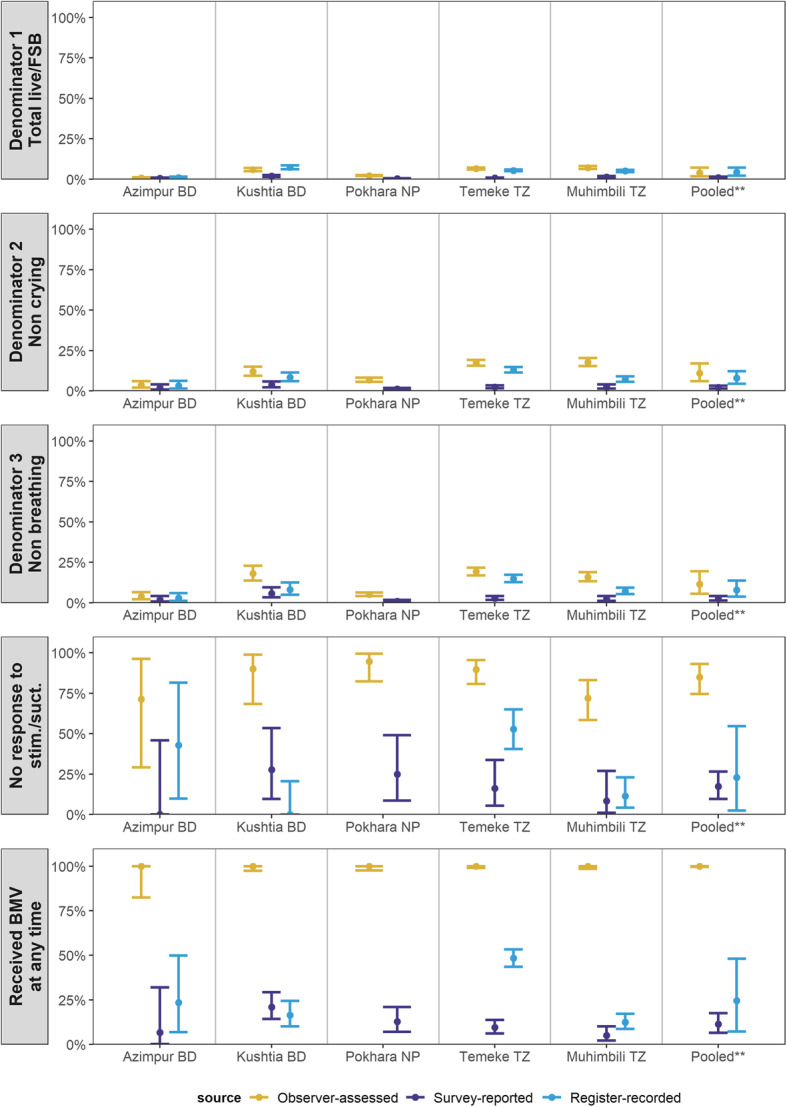
Coverage (and 95%CI) of bag-mask-ventilation measured by observation, register, and exit survey, EN-BIRTH study (*n* = 22,752). *Random effects meta-analysis; BD = Bangladesh, NP = Nepal, TZ = Tanzania, stim. = stimulation, suct. = suction, BMV = bag mask ventilation, FSB = fresh stillbirth; BMV = bag mask ventilation; Full denominator details presented in Additional file 14

Observed BMV ranged from 0.7% in Azimpur BD to 7.1% in Muhimbili TZ. Survey-reported coverage (0.3–1.9%) underestimated observed coverage (Fig. 6). Sensitivity was < 21% while specificity was high across all hospitals (> 98%). Register-recorded coverage (0.9–7.2%) was closer to observed coverage. While sensitivity ranged from 12.4–48.4%, specificity was > 93% across all hospitals (Table 3; additional validity details in Additional files 7 and 8).

**Table 3:** Individual-level validation in registers and exit surveys of bag-mask-ventilation indicator, EN-BIRTH study (n = 22,752)

	Bangladesh	Bangladesh	Nepal	Tanzania	Tanzania	All sites
	Azimpur Tertiary	Kushtia District	Pokhara Regional	Temeke Regional	Muhimbili National	Pooled (random effects)
**D1) Live births and fresh stillbirths**						
**Survey-reported**						
Observer coverage %	0.7 (0.4,1.0)	5.9 (5.0,7.0)	2.1 (1.8,2.5)	6.4 (5.9,7.1)	7.1 (6.3,8.0)	4.0 (1.7,7.0)
Survey reported coverage %	0.5 (0.3,0.9)	1.9 (1.4,2.6)	0.3 (0.2,0.5)	0.7 (0.5,1.0)	1.4 (1.0,1.9)	0.9 (0.5,1.5)
"Don’t know” responses %	3.5 (2.8,4.3)	1.0 (0.7,1.6)	1.6 (1.3,1.9)	0.2 (0.1,0.4)	4.2 (3.5,5.1)	1.8 (0.6,3.6)
Sensitivity % (95% CI)	6.7 (0.2,31.9)	21 (14.2,29.2)	12.9 (7.0,21.0)	9.5 (6.2,13.8)	5.0 (2.0,10.1)	11.3 (6.4,17.5)
Specificity % (95% CI)	99.5 (99.2,99.7)	99.2 (98.7,99.5)	99.9 (99.7,99.9)	99.7 (99.5,99.8)	98.9 (98.3,99.2)	99.5 (99.1,99.8)
Percent agreement (TN + TP/n) %	99.0	94.9	98.4	95.6	93.7	96.6 (94.3,98.4)
**Register-recorded**						
Observer coverage %	0.7 (0.4,1.0)	5.9 (5.0,7.0)	–	6.4 (5.9,7.1)	7.1 (6.3,8.0)	4.5 (1.8,8.4)
Register recorded coverage %	0.9 (0.6,1.5)	7.2 (6.1,8.4)	–	5.4 (4.9,6.0)	5.0 (4.3,5.8)	4.3 (2.1,7.1)
Not recorded %	98.9 (98.3,99.3)	92 (90.7,93.1)	–	9.0 (8.3,9.7)	45.4 (43.8,47.1)	66.0 (15.7,99.3)
Not readable %	0.2 (0.1,0.5)	0.8 (0.5,1.3)	–	0.3 (0.2,0.4)	0.2 (0.1,0.5)	0.3 (0.2,0.6)
Sensitivity % (95% CI)	23.5 (6.8,49.9)	16.4 (10.2,24.4)	–	48.4 (43.6,53.4)	12.4 (8.6,17.2)	24.6 (7.2,48.1)
Specificity % (95% CI)	99.2 (98.8,99.6)	93.4 (92.2,94.4)	–	97.6 (97.2,97.9)	95.5 (94.8,96.2)	96.8 (94.3,98.6)
Percent agreement (TN + TP/n) %	98.7	89.0	–	94.4	89.7	93.6 (88.8,97.2)
**D2) Non-crying**						
**Survey-reported**						
Observer coverage %	3.6 (2.1,6.2)	12.0 (9.5,15.1)	6.8 (5.7,8.2)	17.4 (15.7,19.2)	17.8 (15.4,20.4)	10.9 (6.1,17.0)
Survey reported coverage %	2.0 (0.9,4.3)	3.8 (2.3,5.9)	1.2 (0.7,2)	2.5 (1.7,3.4)	2.5 (1.5,4.2)	2.3 (1.5,3.3)
"Don’t know” responses %	9.7 (6.9,13.4)	1.8 (0.9,3.5)	3.7 (2.8,4.9)	0.7 (0.4,1.3)	8.0 (6.0,10.6)	4.1 (1.5,8.0)
Sensitivity % (95% CI)	9.1 (0.2,41.3)	16.1 (8.0,27.7)	14.7 (7.3,25.4)	10.8 (6.8,16.0)	7.4 (3.0,14.7)	11.6 (8.7,14.8)
Specificity % (95% CI)	98.2 (96.2,99.3)	98 (96.2,99.1)	99.5 (99,99.8)	98.9 (98.1,99.4)	98.4 (96.9,99.3)	98.7 (98.0,99.3)
Percent agreement (TN + TP/n) %	95.4	87.9	95.3	86.8	84.1	90.4 (85.1,94.6)
**Register-recorded**						
Observer coverage %	3.6 (2.1,6.2)	12 (9.5,15.1)	–	17.4 (15.7,19.2)	17.8 (15.4,20.4)	12.1 (6.9,18.5)
Register recorded coverage %	3.3 (1.6,6.4)	8.5 (6.2,11.5)	–	13 (11.5,14.7)	7.2 (5.7,9.1)	7.9 (4.4,12.2)
Not recorded %	96.7 (93.6,98.4)	90.9 (87.8,93.3)	–	10.1 (8.8,11.7)	47.1 (43.9,50.4)	64.4 (19.2,97.4)
Not readable %	0.0 (0.0,1.7)	0.7 (0.2,2.1)	–	0.3 (0.1,0.7)	0 (0,0.5)	0.2 (0.0,0.6)
Sensitivity % (95% CI)	25.0 (5.5,57.2)	11.1 (3.7,24.1)	–	46.8 (41.1,52.5)	13.5 (8.7,19.7)	23.7 (6.5,47.2)
Specificity % (95% CI)	97.7 (95.1,99.2)	91.8 (88.8,94.3)	–	94.1 (92.8,95.3)	94.1 (92.2,95.7)	94.4 (92.4,96.1)
Percent agreement (TN + TP/n) %	94.5	83.9	–	85.9	80.0	86.3 (81.1,90.8)
**D3) Non-breathing**						
**Survey-reported**						
Observer coverage %	3.9 (2.2,6.7)	18.0 (13.8,23)	5.1 (4.1,6.4)	19.2 (16.9,21.7)	15.9 (13.3,18.9)	11.5 (5.5,19.5)
Survey reported coverage %	1.9 (0.8,4.3)	5.8 (3.4,9.6)	1.0 (0.5,1.8)	2.7 (1.7,4.1)	2.3 (1.2,4.3)	2.5 (1.3,4.1)
"Don’t know” responses %	10.6 (7.5,14.7)	2.3 (0.9,5.2)	3.2 (2.3,4.4)	0.6 (0.2,1.6)	7.1 (5.0,10.0)	4.1 (1.5,7.9)
Sensitivity % (95% CI)	0.0 (0.0,30.8)	20.8 (10.5,35.0)	16.7 (7.0,31.4)	11.3 (6.2,18.6)	8.2 (2.7,18.1)	12.8 (8.0,18.5)
Specificity % (95% CI)	98.0 (95.7,99.3)	97.6 (94.5,99.2)	99.6 (99.0,99.9)	98.8 (97.7,99.5)	98.7 (96.9,99.6)	98.7 (97.8,99.4)
Percent agreement (TN + TP/n) %	94.9	83.3	96.7	86.1	86.0	90.1 (83.1,95.4)
**Register-recorded**						
Observer coverage %	3.9 (2.2,6.7)	18.0 (13.8,23.0)	–	19.2 (16.9,21.7)	15.9 (13.3,18.9)	13.6 (7.5,21.1)
Register recorded coverage %	2.9 (1.3,6.1)	8.2 (5.1,12.7)	–	14.9 (12.8,17.3)	7.1 (5.4,9.4)	8.0 (3.7,13.7)
Not recorded %	97.1 (93.9,98.7)	90.5 (85.8,93.8)	–	9.7 (8.0,11.8)	46.6 (42.8,50.4)	64.2 (19.3,97.2)
Not readable %	0.0 (0.0,1.9)	1.3 (0.3,4.0)	–	0.2 (0.0,0.8)	0.0 (0.0,0.7)	0.2 (0.0,0.7)
Sensitivity % (95% CI)	25.0 (5.5,57.2)	11.1 (3.1,26.1)	–	51.3 (44,58.5)	15.7 (9.4,24)	25.6 (7.2,50.2)
Specificity % (95% CI)	98.3 (95.6,99.5)	92.3 (87.7,95.7)	–	93.8 (92,95.4)	94.5 (92.3,96.2)	94.8 (92.5,96.7)
Percent agreement (TN + TP/n) %	94.6	79.7	–	85.6	82.1	85.9 (80.2,90.8)

Full denominator details presented in Additional file 14

### Objective 2: Denominator for indicator measurement comparison

#### Denominator option 1: all newborns (live births and fresh stillbirths)

**Fig. 7:**
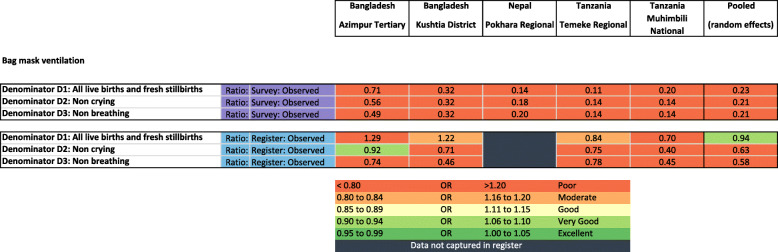
Validity ratios for exit survey-reported and register-recorded coverage of bag-mask-ventilation, EN-BIRTH study (*n* = 22,752). Full denominator details presented in Additional file 14

The validation of birth outcomes is reported separately [28]. Survey validity ratios for BMV coverage measurement using this all newborn denominator performed poorly (0.11–0.71) and register validity ratios were moderate to poor (0.70–1.22) (Fig. 7).

#### Denominator option 2: non-crying newborns

Survey-reported prevalence of crying at birth (90.5–95.8%) was higher than observed prevalence of crying within 1 min of birth (72.0–86.7%) with very few “don’t know” responses (< 3%). While sensitivity was high (> 95%) specificity was low (< 22%) (Table 4; additional validity details in Additional files 9 and 10).

**Table 4:** Individual-level validation in exit survey of crying at birth indicator, EN-BIRTH study (n = 22,752 births)

	Bangladesh	Bangladesh	Nepal	Tanzania	Tanzania	All sites
	Azimpur Tertiary	Kushtia District	Pokhara Regional	Temeke Regional	Muhimbili National	Pooled Random effects
**Cry at birth - Survey reported – live births + fresh stillbirths**						
Observer prevalence %	86.7 (85.3,87.9)	75.7 (73.8,77.4)	77.4 (76.4,78.3)	72.0 (70.9,73)	72.6 (71.1,74.1)	77.1 (72.0,81.8)
Survey reported prevalence %	94.4 (93.4,95.2)	94.3 (93.3,95.2)	95.8 (95.3,96.3)	93.0 (92.3,93.6)	90.5 (89.3,91.6)	93.7 (91.9,95.3)
"Don’t know” responses %	0.8 (0.5,1.2)	1.2 (0.8,1.7)	1.3 (1.0,1.6)	0.8 (0.6,1.0)	2.6 (2.0,3.3)	1.3 (0.8,1.8)
Sensitivity % (95% CI)	96.0 (95.1,96.7)	98.5 (97.8,99.0)	97.3 (96.8,97.7)	97.7 (97.2,98.1)	95.4 (94.3,96.3)	97.1 (96.1,97.9)
Specificity % (95% CI)	15.7 (12.2,19.8)	15.2 (12.3,18.5)	8.7 (7.3,10.2)	18.1 (16.2,20.2)	21.4 (18.3,24.8)	15.6 (10.8,21.0)
Percent agreement (TN + TP/n) %	85.3	78.4	77.0	76.6	76.3	78.8 (75.6,81.9)

Full denominator details presented in Additional file 14

Survey validity ratios for BMV using this non-crying denominator performed poorly (0.13–0.58), while sensitivity was low (< 16%), specificity was high (> 98%). Register validity ratios ranged from poor to very good (0.40–0.92). While sensitivity was low (11.1–46.8%), specificity was high (> 91%).

#### Denominator option 3: non-breathing newborns

Prevalence of not breathing within the first minute ranged from 11.7% in Azimpur BD to 21.0% in Pokhara NP. The survey validity ratio for BMV coverage measurement using this non-breathing denominator performed poorly (0.14–0.49). Sensitivity ranged from 0 to 20.8% while specificity was > 97% across hospitals. Register validity ratios were better, but still classified as poor (0.45–0.78). While sensitivity ranged from 11.1–51.3%, specificity was high across all hospitals (> 92%).

### Objective 3: Coverage and quality gap analysis

**Fig. 8:**
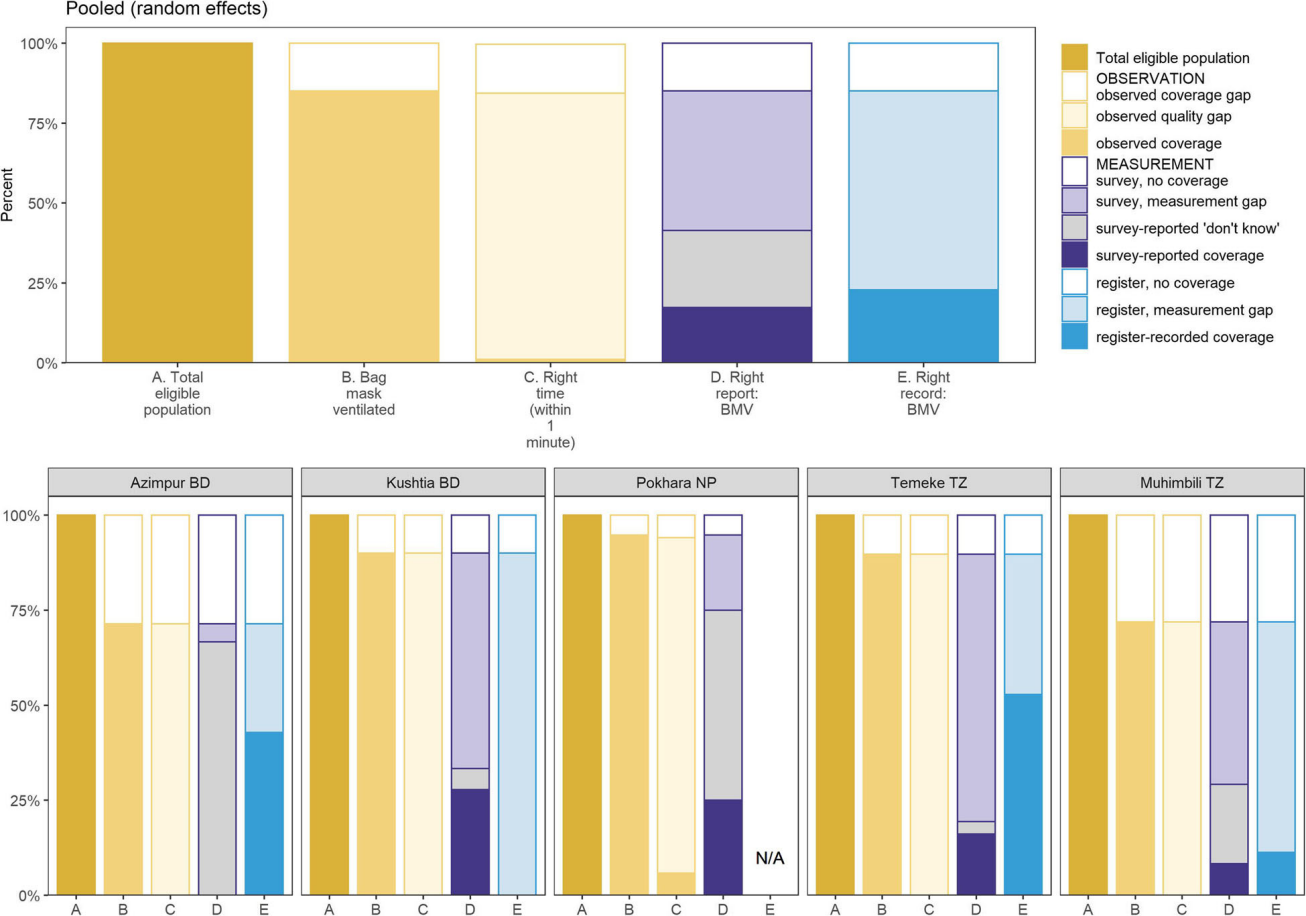
Gap analysis for coverage and quality among newborns non-crying/not responding to stimulation/suction, EN-BIRTH study (*n* = 200). BD = Bangladesh, NP = Nepal, TZ = Tanzania; BMV = Bag-mask-ventilation; Full denominator details presented in Additional file 14

Among the subset proxy for true clinical need [newborns who did not cry/breathe in the first minute with no response to stimulation (or suction if needed)], most received BMV, ranging from 71.4% in Azimpur BD to 94.7% in Pokhara NP (Fig. 8) but timely coverage was very low (1%). Survey-reported coverage (< 28%) substantially underestimated true coverage. Register-recorded coverage also underestimated true coverage and ranged widely from 0.0% in Kushtia BD to 52.9% in Temeke TZ.

**Fig. 9:**
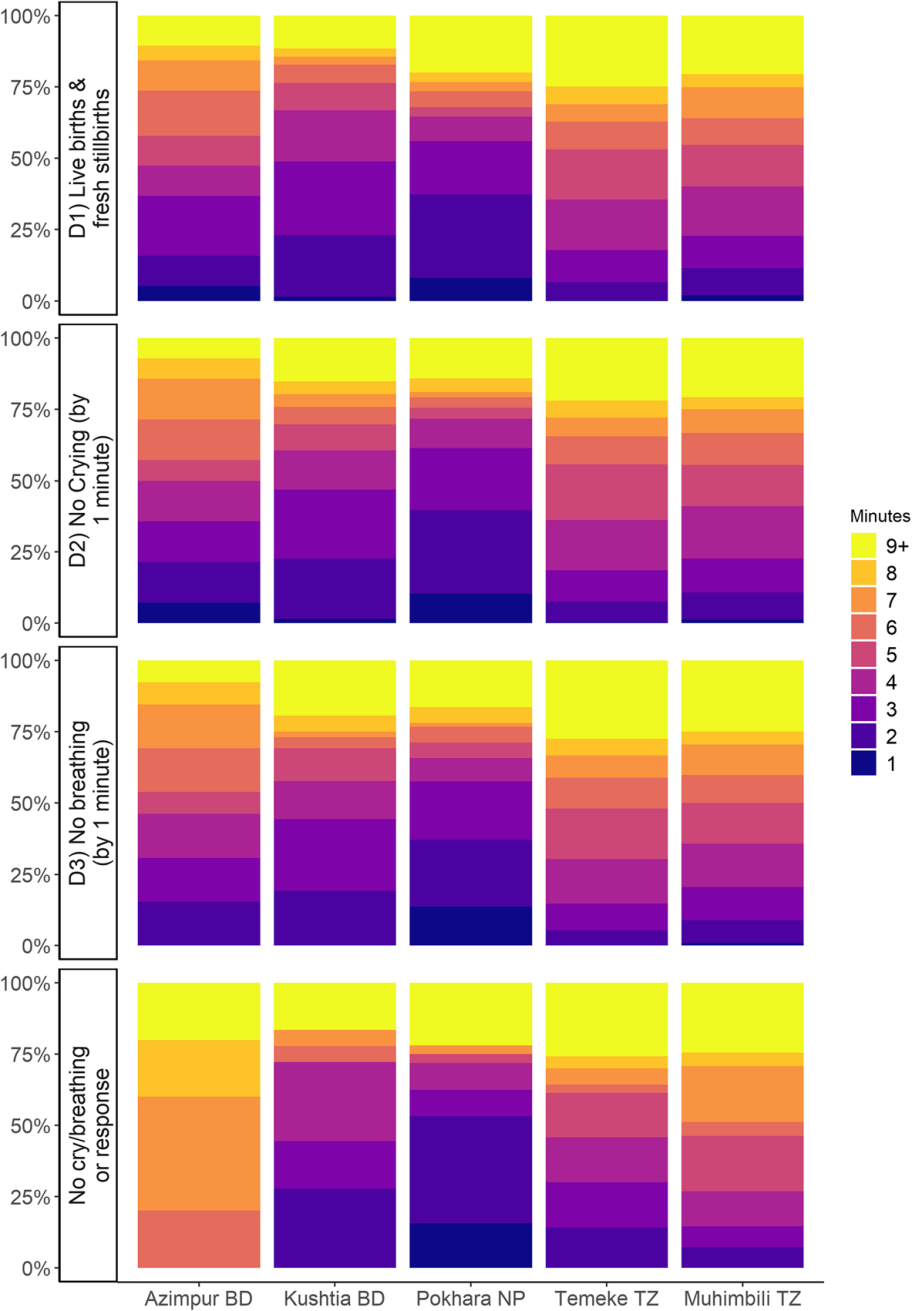
Time to bag-mask-ventilation by denominator, EN-BIRTH study (*n* = 991). BD = Bangladesh, NP = Nepal, TZ = Tanzania; D1: n = 991, D2: *n* = 672, D3: *n* = 454, No cry/breath/response: *n* = 142

Among newborns receiving any BMV on the labour ward, the proportion receiving the first ventilation breath within 1 min of birth ranged from 0.2% in Temeke TZ to 8.0% in Pokhara NP. Across the three denominators explored, time to initiation of BMV was similar (Fig. 9).

### Objective 4: Barriers and enablers to routine recording

#### Register design

Labour ward registers varied in design, between the five hospitals (Fig. 5). Bangladesh labour ward registers had three specific columns for recording neonatal resuscitation: (i) “baby did not breathe/cry after birth” (tick box for ‘yes’ and tick box for ‘no’), (ii) “stimulation” (instructions to tick for ‘yes’ and leave blank for ‘no’) and (iii) “BMV” (instructions to tick for ‘yes’ and leave blank for ‘no’). The Tanzanian register captured resuscitation steps by numerical code in a column headed “Helping Babies Breathe” (suction = 1, stimulation = 2, BMV = 3) or “no”, and blanks are treated as not recorded. There was no specific column in the Nepal register for resuscitation.

#### Documentation practices in registers

Resuscitation practices were recorded in varying order into multiple documents (Additional file 11). Reported time between care and documentation ranged from 2.5 min in Pokhara NP to 22.5 min in Temeke TZ.

##### Register design

Register design largely acted as a barrier to recording in Pokhara NP:

*“Drying, stimulation, and bag-mask ventilation are written [in the patient’s chart], but in the main register it is not present… we do not have routine care of the newborn in the register, only in the patient’s chart.”*

-Data collector, Pokhara NP

In the other hospitals health workers duplicated documentation in registers with multiple other documents (e.g. partographs, patient case notes) (Additional file 12).

##### Register filling

Aspects of register filling acted as both barriers and enablers. Training and support from senior nurses enabled improved accuracy of documentation, while limited time acted as a barrier. Health workers across the hospitals discussed the lack of time to document, particularly for complicated cases and resuscitation when they are focused on delivering care:

*“Just after finishing [resuscitation], you must keep everything clear… time is a problem… you must estimate, there are times it is difficult and other times you ask the [senior nurse]… because in an emergency you all work together; thus, you remind each other.”*

– Health worker, Temeke TZ

Health workers in Pokhara NP received specific support for documentation in neonatal resuscitation:

*“We have received training on HBB and we were trained for documentation in that. We were doing documentation before, but we received direction for improving it.”*

-Health worker, Pokhara NP

However, while health workers in Pokhara NP record resuscitation in other documents, it is not recorded in routine hospital registers.

##### Register use

While improved patient care and use of data by managers motivated documentation and was affirmation of the care health workers were giving, not all respondents could identify the use for resuscitation data in routine registers.

Feedback was lacking where documentation didn’t line up with clinical need:

*“Sometimes when you look at the [APGAR] score of the baby, maybe it’s 5, you wonder why they didn’t perform resuscitation, there’s a possibility they [did] but they haven’t documented that… There’s no one to follow up on that… The person responsible for data comes and copies what’s written in the register, be it a low score… but they never ask them why they didn’t perform resuscitation if the baby had a low score”*

– Health worker, Temeke TZ

Conversely, in Bangladesh, health workers were not sure what happened with resuscitation data:

*“Resuscitation is an emergency subject. There remains a referral slip while resuscitating a baby on emergency that indicates the baby went to operating theatre... We write down the procedures of resuscitation in that slip... I am not sure whether this actually goes in the monthly report or not.”*

-Health worker, Azimpur BD

##### Data culture

Data culture was both an enabler and barrier to routine documentation of resuscitation. It acted as a barrier where minor interventions were not seen as worth recording:

*“Minor things like suctioning were not recorded and they only documented on a resuscitation case that took more than ten minutes.”*

-Data collector, Muhimbili TZ

However, the importance of documentation was noted for organizational and personal protection:

*“For instance, if a child has been born but unfortunately, let us say she had a problem, you have resuscitated her, but you did not document… and the mother/parent has become very angry and start complaining, or the whole management has become angry with you why the child had this situation, but you did not record what you have done ... You will not defend yourself, but documentation defends you.”*

-Health worker, Muhimbili TZ

## Discussion

EN-BIRTH study’s large sample size (22,752 live births and fresh stillbirths) allowed the first validity assessment of measurement for neonatal resuscitation coverage in routine hospital registers and surveys, against a gold standard of clinical observation. We found that survey report poorly captured resuscitation indicators. Routine labour ward registers performed better, but variably, and have potential, especially with data quality improvement.

Survey-reported coverage was challenging, which is not surprising. We found most women who reported their baby had trouble breathing after birth did not know if their baby had been stimulated or received BMV. We recommend resuscitation need or BMV questions should not be added to existing population-based surveys. Furthermore, the sample size required for this relatively low-incidence practice, would be challenging even in DHS surveys with large, nationally representative samples [29].

The numerator for neonatal resuscitation is key. Stimulation by rubbing the baby’s back is easily conflated with the similar action of drying every newborn baby and was not recognized at all by mothers (< 3% in survey report). Suction is only necessary if the airway is blocked and a measurement focus on suction may unintentionally encourage this potentially harmful practice which can cause bradycardia. BMV is the most distinguishable option for a clear subset of non-breathing babies and had higher accuracy than stimulation. Though underestimated in surveys, accuracy of BMV was still performed better than stimulation by survey-report. Additionally, BMV is a more suitable intervention for which to assess quality and links to health facility assessments where standard questions include presence and recent use of neonatal bag and masks.

Health facilities are where ~ 80% of women now deliver [10], providing an opportunity to track neonatal resuscitation coverage through routine facility data using BMV as the numerator. Four of the five routine registers assessed were already capturing BMV count data. At the population level, register-recorded coverage of BMV was within 2.1% of observed coverage although individual-level validation metrics suggested low sensitivity. Selective register design is important in capturing what is needed yet avoiding documentation over-burdening. In Tanzania, the register column labelled “HBB” aligns measurement with scale-up programming. The design in Bangladesh instructed health workers to leave the column blank when BMV is not done; thus, calculating completeness and differentiating between truly ‘not done’ and register ‘incomplete’ was impossible. Where register instructions in Tanzania state to write “no” if BMV was not done, completeness was moderate to high (54.6–91.0%). Although data collectors rarely indicated data were not readable (< 0.5%), there were low inter-rater kappa results for register-recorded BMV in some sites [23]. Because extraction/aggregation is the first step for data flowing to higher levels in the health system, more research is needed to improve this. Capturing reliable data depends on user-friendly, appropriate recording systems, however, accuracy varied even within the same country using identical register design, highlighting the importance of information culture and supervision. Our qualitative findings suggest differences in understanding of importance and utility of resuscitation data at different hospitals.

Denominators are notably challenging for interventions such as resuscitation which are indicated based on clinical need for only a subset of babies [30]. Current WHO guidance recommends *number of live births in a facility*, with a footnote that this is pragmatic whilst ongoing work to test different denominators, including EN-BIRTH, is completed [31]. Here we have included live births plus fresh stillbirths, for whom resuscitation is recommended. Any newborn without maceration or major malformations, even if they appear completely lifeless, should be given the chance of resuscitation [32]. The reduction in stillbirth rates associated with resuscitation training [33–35] are likely results of reduced misclassification of live births as stillbirths.

Measuring the true denominator for clinical need for resuscitation is complex. Newborns require BMV if non-breathing/gasping after initial drying/stimulation or if they suffer subsequent apnoea at any time. Breathing well may be difficult to measure as the concept excludes gasping, fast breathing and grunting. It is critical to emphasise these breathing patterns during clinical training as BMV is indicated for some (e.g. gasping) but not all of these breathing patterns. EN-BIRTH observers collected breathing or not breathing as a binary variable because formative research suggested other breathing patterns were not feasible to capture. In our study, 2/5 registers captured non-breathing but as a composite non-crying and non-breathing indicator. Consequently, accuracy of this denominator in registers could not be assessed.

Non-crying has potential utility as a denominator as it is simple for health workers to capture and is part of the process in assessing need for resuscitation. Additionally, crying at birth is a single event and thus more straightforward to record as opposed to breathing which is a process and might change over time, particularly for preterm babies. While not all non-crying babies will require further steps of resuscitation, almost all babies who do need BMV are non-crying. One study has shown babies breathing but not crying after birth have an increased risk of death [36]. We found the observed coverage of BMV ranged from 3.6–17.8% among babies not crying in the first minute. Further research is required to assess if non-crying is useful and benchmarking is feasible. However, as considerations turn towards respectful newborn care and minimal handling, further research is needed related to newborn physiological responses after birth and what is appropriate to measure.

Apgar scores are captured in all the routine hospital registers in our study, including in Pokhara NP, which captured no resuscitation interventions. Apgar scores do not capture interventions around the time of birth, rather describe a newborn’s physical condition and response to any interventions at 1 and 5 min after birth and are already known to have limitations, notably low inter-rater reliability. The one-minute Apgar score, which includes heart rate, does not fit well with current resuscitation algorithms which recommend checking the baby’s heart rate after a minute of ventilation (2 min after birth). As such, the Apgar score is not a useful denominator for neonatal resuscitation and as usually written in individual patient records, we suggest exploring replacing this column in routine labour ward registers with data elements that can be used for coverage measurement e.g. not crying after birth.

Timely resuscitation is essential and even small delays in starting resuscitation can contribute to death or disability [37]. Our assessment of quality of care focused on timeliness of the start of BMV within the first minute after birth. While coverage of BMV was high (85%), only 1% of newborns received the first ventilation within 1 min of birth. In the all newborns denominator, not all will require BMV within 1 min of birth as many were crying/breathing at birth and subsequently became distressed or apnoeic. A coverage gap for BMV of fresh stillbirths is to be expected as it is not appropriate to resuscitate those babies who are diagnosed before birth to have died in utero e.g. confirmed by ultrasound. Measuring timing of BMV is clearly not feasible in surveys and very unlikely to be possible in routine labour ward registers. Given this major quality gap regarding timing of resuscitation initiation, local audit and special studies are important to drive quality improvement.

### Strengths and limitations

Strengths of this study include the multi-site and multi-country design and large sample size enabling the capture of multiple decision points on resuscitation algorithms. We evaluated how several possible numerators/denominators performed using clinical observation as a gold standard. We assessed possible bias in the observation data with double observation for a subset of cases. Overall, BMV had good inter-observer agreement. Whilst clinically trained observers provided gold standard data on coverage of interventions, subjectivity remains possible e.g. differentiating stimulation from immediate drying. To limit this, the tablet application was designed to capture stimulation in a specific neonatal resuscitation section separate from the immediate care practices, such as drying. The low coverage of stimulation amongst non-crying/breathing newborns (34–38%) may reflect poor quality of care or difficulty in measurement for stimulation by an observer.

Some other limitations should be noted. Survey-reported coverage was assessed in exit survey, closer in time to the events in question than standard population-based surveys with 2–5-year reference periods. In survey, only women who answered ‘yes’ to a question asking whether their baby had difficulty breathing at birth were asked further questions about resuscitation, thus some who may have recognised newborn stimulation were not counted towards survey-reported coverage. Additionally, the EN-BIRTH study sample may be healthier than the average in these facilities (women too sick to consent, women with no fetal heart beat heard at admission, etc., were excluded from the study). As the study sites were CEmONC hospitals, case mix, coverage, and measurement may differ at lower-level facilities.

Importantly, the true denominator of babies in need of BMV will not be captured by facility measurement, especially the disadvantaged who are more likely to deliver at home in LMICs. However, home births are less likely to receive BMV in most LMICs, so facility measurement is likely to capture nearly all the numerator in terms of newborns receiving BMV. Hence approaches such as those used in immunisation when the denominator is missing may help to estimate the coverage of the whole population for contexts with many home births.

## Conclusion

Neonatal resuscitation is a high impact evidence-based intervention for a leading cause of under-five mortality, preventable stillbirth and disability. Yet the current lack of coverage measurement is impeding global tracking of scale-up in high-burden countries. We found bag-mask-ventilation was the most reliable numerator. Measuring the true denominator for clinical need is complex and further denominator research is required, including respectful care considerations, evaluating non-crying as a potential alternative. Based on these results, we do not recommend tracking this indicator through population-based survey. Register measurement of neonatal resuscitation has potential and if standardised and included in HMIS, could aid in tracking progress towards global targets across countries. An appropriate resuscitation denominator could potentially replace the Apgar score, which was recorded as a column in all five registers. Implementation research is needed regarding how to improve register data quality. Measuring and addressing quality of care gaps, notably for timely provision of resuscitation in the first minute, is crucial for programme improvement and impact, but unlikely to be feasible in routine systems, requiring audits and special studies. Improving data is possible and necessary, informing progress to meet global goals and meet every family’s aspiration that their baby will survive and thrive.

## Supplementary Information

**Additional file 1.** Data collection dates by site for the EN-BIRTH study.

**Additional file 2.** EN-BIRTH metadata definitions of selected indicator options for validity testing.

**Additional file 3.** STROBE statement—checklist of items that should be included in reports of observational studies.

**Additional file 4.** EN-BIRTH data quality assurance for Gold standard – double observation and data entry.

**Additional file 5.** Validity measures for stimulation (AUC, IF, PPV, NPV) by hospital for EN-BIRTH study.

**Additional file 6.** EN-BIRTH study – testing validity – stimulation coverage indicator 2 way tables.

**Additional file 7.** Validity measures for BMV (AUC, IF, PPV, NPV) by hospital and denominator option for EN-BIRTH study.

**Additional file 8.** EN-BIRTH study – testing validity – BMV coverage indicator 2 way tables.

**Additional file 9.** Validity measures for survey-reported crying at birth by hospital for EN-BIRTH study.

**Additional file 10.** EN-BIRTH study – testing validity – cry at birth 2 way tables.

**Additional file 11.** Recording order of neonatal resuscitation in hospital documents according to EN-BIRTH data collectors.

**Additional file 12.** Barriers and enablers to routine recording of neonatal resuscitation.

**Additional file 13.** Ethical approval of local institutional review boards, EN-BIRTH study.

**Additional file 14.** Numerators and denominators for observed, survey-reported and register-recorded indicators.

## Data Availability

The datasets generated during and/or analysed during the current study are available on LSHTM Data Compass repository, https://datacompass.lshtm.ac.uk/955/.

## Abbreviations

EN-BIRTH: Every Newborn–Birth Indicators Research Tracking in Hospitals
BD: Bangladesh
BMV: Bag-and-mask ventilation
CEmONC: Comprehensive Emergency Obstetric and Neonatal Care
DHS: Demographic and Health Survey Program
HBB: Helping Babies Breathe
HMIS: Health Management Information Systems
icddr,b: International Centre for Diarrheal Disease Research, Bangladesh
IHI: Ifakara Health Institute
LMIC: Low- and middle-income country
LSHTM: London School of Hygiene & Tropical Medicine
MCHTI: Maternal and Child Health Training Institute, Azimpur, Bangladesh
MICS: Multiple Indicator Cluster Survey
MUHAS: Muhimbili University of Health and Allied Sciences
NP: Nepal
TZ: Tanzania
WHO: World Health Organization
