# Supplementary material for: Neonatal resuscitation: EN-BIRTH multi-country validation study
Source: BMC Pregnancy Childbirth. 2021 Mar 26;21(Suppl 1):235. doi: 10.1186/s12884-020-03422-9 (PMC7995695; doi:10.1186/s12884-020-03422-9)

Every Newborn BIRTH multi−country validation study: informing measurement of coverage and quality of maternal and newborn care

Neonatal resuscitation: EN−BIRTH multi−country validation study

Additional file 11. Recording order of neonatal resuscitation in hospital documents according to EN−BIRTH data collectors.

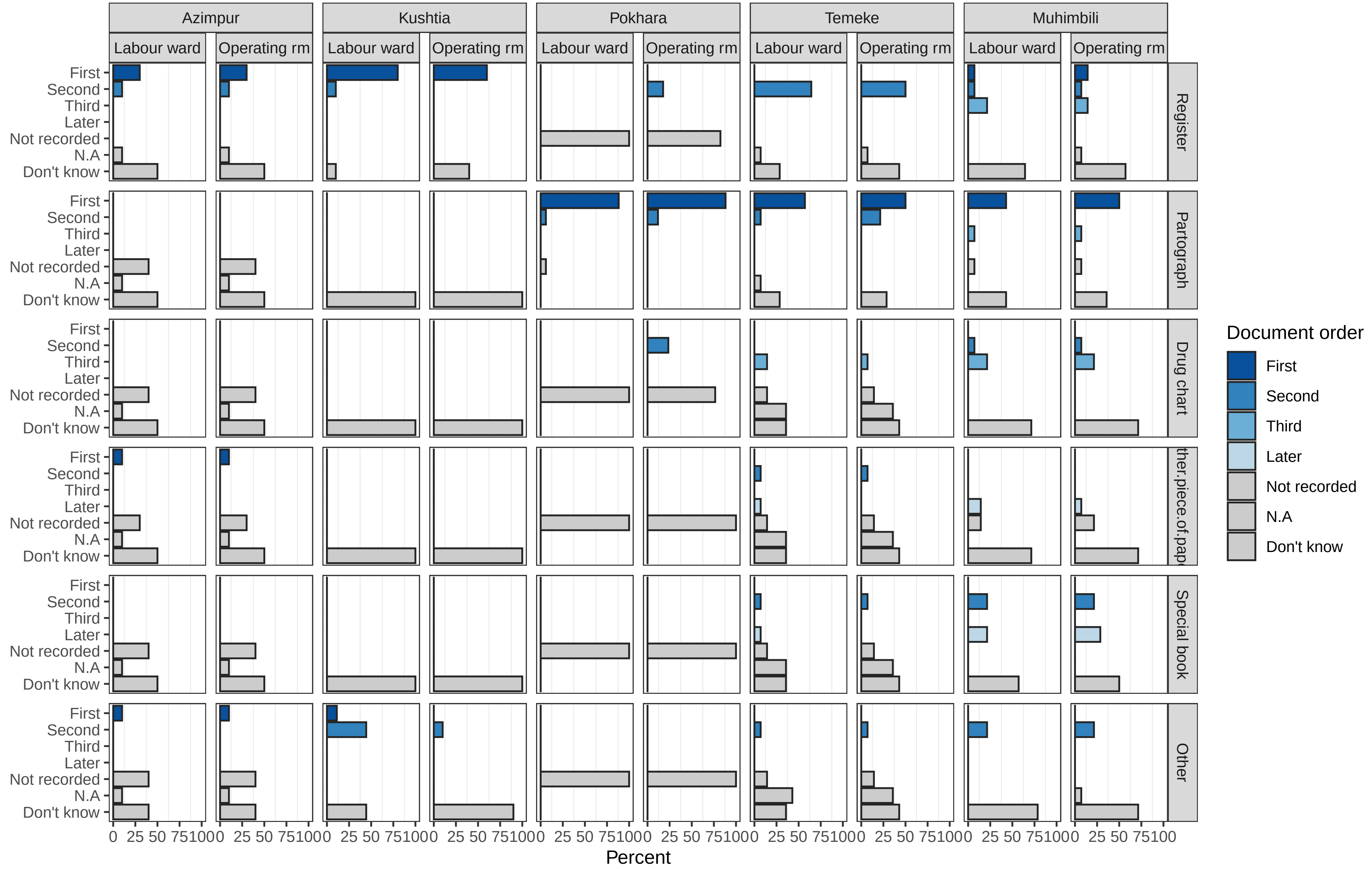

Supplement: Supplementary file 11 — Additional file 11. Recording order of neonatal resuscitation in hospital documents according to EN-BIRTH data collectors. [file 12884_2020_3422_MOESM11_ESM.pdf]
