# Supplementary material for: Neonatal resuscitation: EN-BIRTH multi-country validation study
Source: BMC Pregnancy Childbirth. 2021 Mar 26;21(Suppl 1):235. doi: 10.1186/s12884-020-03422-9 (PMC7995695; doi:10.1186/s12884-020-03422-9)
Supplement: Supplementary file 12 — Additional file 12. Barriers and enablers to routine recording of neonatal resuscitation. [file 12884_2020_3422_MOESM12_ESM.pdf]

Every Newborn BIRTH multi-country validation study: informing measurement of coverage and quality of maternal and newborn care

Neonatal resuscitation: EN-BIRTH multi-country validation study

Additional File 12: Barriers and enablers to routine recording of neonatal resuscitation, EN-BIRTH study

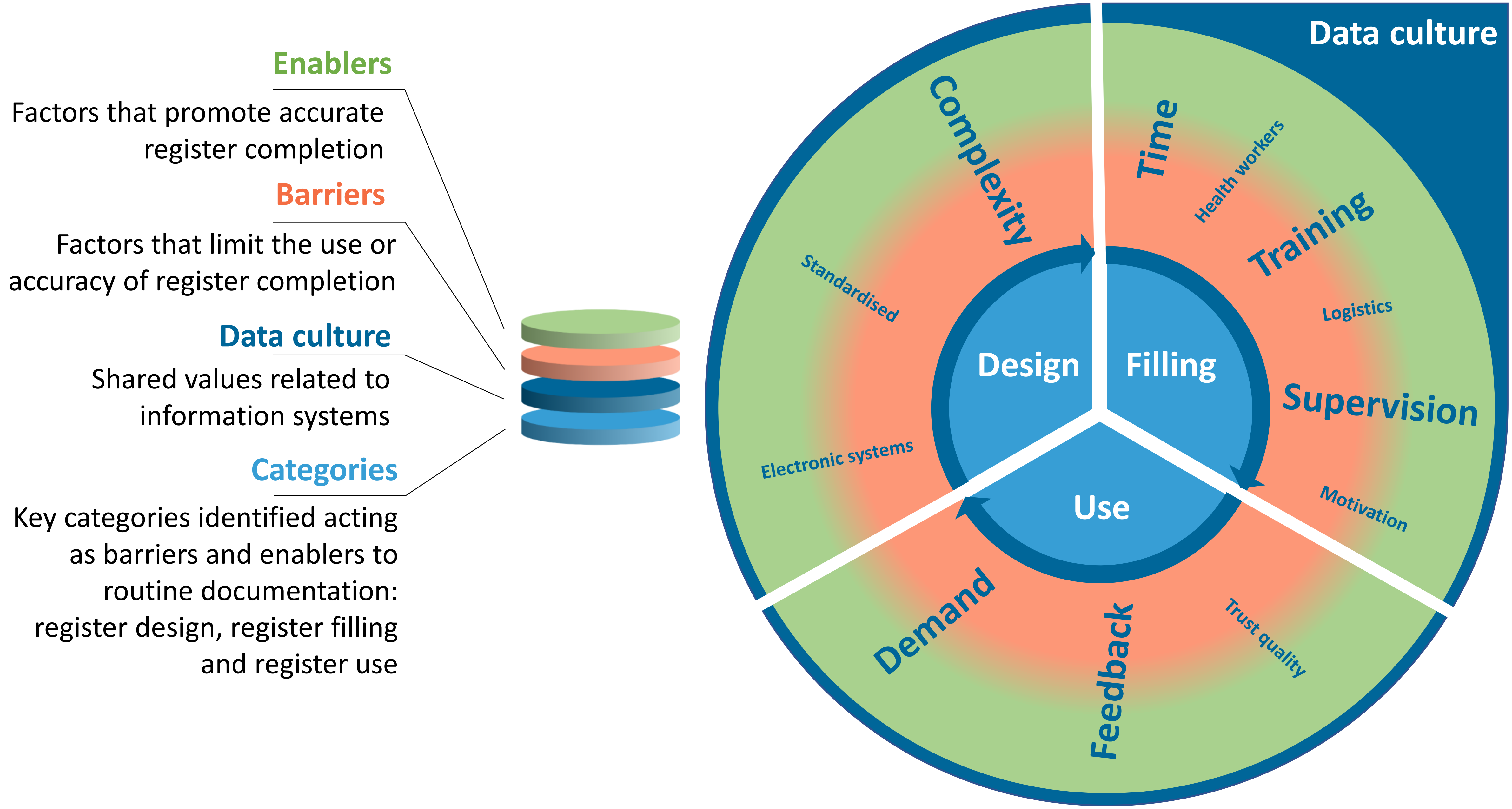

This figure illustrates the overall barriers and enablers to facility-based data collection identified by EN-BIRTH participants. The bold text are the issues specific to resuscitation. The transition from red to green is a reminder that most factors identified by participants could serve as either a barrier or enabling factor depending on the facility-level resources and management
