# Supplementary material for: Neonatal resuscitation: EN-BIRTH multi-country validation study
Source: BMC Pregnancy Childbirth. 2021 Mar 26;21(Suppl 1):235. doi: 10.1186/s12884-020-03422-9 (PMC7995695; doi:10.1186/s12884-020-03422-9)
Supplement: Supplementary file 13 — Additional file 13. Ethical approval of local institutional review boards, EN-BIRTH study. [file 12884_2020_3422_MOESM13_ESM.pdf]

Every Newborn BIRTH multi-country validation study: informing measurement of coverage and quality of maternal and newborn care

Neonatal resuscitation: EN-BIRTH multi-country validation study

Additional File 13: Ethical approval of local institutional review boards, EN-BIRTH study

Country	Institutional Review Boards	Date	Number/Ref
UK	London School of Hygiene & Tropical Medicine (LSHTM) Interventions Research Ethics Committee	03/10/16	11780
Bangladesh	Icddr,b Research review Committee<br>Icddr,b ethical review committee	11/08/16<br>14/11/16	PR 16055
Nepal	Nepal Health Research Council (NHRC)	08/08/16	187/2016
		15/03/17	26/2017
		13/02/19	95/2019
Tanzania	National Institute for Medical Research (NIMRI)<br>Ifakara Health Institute<br>Muhimbili University of Health and Allied Sciences research and Publications committee	20/01/17<br>20/10/16<br>21/10/16	NIMR/HQ/R.8a/Vol IX/2394<br>IHI/IRB/No: 032-2016<br>2016-10-21-/AEC/Vol.XI/310

Voluntary informed consent was obtained from all participants and their care providers. All women were provided with a description of the study procedures in their preferred language at admission, and offered the right to refuse, or withdraw consent at any time during the study. Facility staff were identified before data collection began and approached for recruitment and consent. No health worker refused participation and all maintained the right to withdraw throughout the study.

This study was granted ethical approval by institutional review boards in all operating counties in addition to the London School of Hygiene & Tropical Medicine.
